# Targeting FGFR for cancer therapy

**DOI:** 10.1186/s13045-024-01558-1

**Published:** 2024-06-03

**Authors:** Pei Zhang, Lin Yue, QingQing Leng, Chen Chang, Cailing Gan, Tinghong Ye, Dan Cao

**Affiliations:** 1 Division of Abdominal Tumor Multimodality Treatment, Cancer Center, West China Hospital, Sichuan University, No. 37 Guoxue Alley, Chengdu, 610041 Sichuan China; 2 Laboratory of Gastrointestinal Cancer and Liver Disease, Department of Gastroenterology and Hepatology, State Key Laboratory of Biotherapy, West China Hospital, Sichuan University, Chengdu, 610041 China

**Keywords:** FGFR, Tumors, Clinical trials, FDA-approved drugs, Drug resistance

## Abstract

The FGFR signaling pathway is integral to cellular activities, including proliferation, differentiation, and survival. Dysregulation of this pathway is implicated in numerous human cancers, positioning FGFR as a prominent therapeutic target. Here, we conduct a comprehensive review of the function, signaling pathways and abnormal alterations of FGFR, as well as its role in tumorigenesis and development. Additionally, we provide an in-depth analysis of pivotal phase 2 and 3 clinical trials evaluating the performance and safety of FGFR inhibitors in oncology, thereby shedding light on the current state of clinical research in this field. Then, we highlight four drugs that have been approved for marketing by the FDA, offering insights into their molecular mechanisms and clinical achievements. Our discussion encompasses the intricate landscape of FGFR-driven tumorigenesis, current techniques for pinpointing FGFR anomalies, and clinical experiences with FGFR inhibitor regimens. Furthermore, we discuss the inherent challenges of targeting the FGFR pathway, encompassing resistance mechanisms such as activation by gatekeeper mutations, alternative pathways, and potential adverse reactions. By synthesizing the current evidence, we underscore the potential of FGFR-centric therapies to enhance patient prognosis, while emphasizing the imperative need for continued research to surmount resistance and optimize treatment modalities.

## Background

The Fibroblast Growth Factor Receptor (FGFR) comprises a subset of Receptor Tyrosine Kinases (RTKs), encompassing five members (*FGFR* 1–5) and exhibiting significant sequence homology [1]. Notably, *FGFR* 5 (also recognized as *FGFRL* 1) lacks the tyrosine kinase domain yet assumes a role in modulating excessive activation of the FGF-*FGFR* signaling pathway [2–4].

FGFR signaling predominantly entails Fibroblast Growth Factor (FGF) binding to the receptor, receptor dimerization, and subsequent intracellular kinase auto-phosphorylation cascades [5]. Additionally, *FGFR* can undergo ligand-independent activation, exemplified by fusion events between the *FGFR* gene and other gene components initiated by chromosomal translocation [6]. The FGF/FGFR signaling pathway intricately intersects embryonic development, angiogenesis, tissue homeostasis, and wound healing, while concurrently orchestrating pivotal functions in cellular proliferation, differentiation, apoptosis, and migration [7, 8]. Nevertheless, the abnormalities of FGF/FGFR signaling axis can promote a variety of diseases, especially malignant tumors, mainly due to the occurrence of gene amplification, mutation, and gene fusion [9, 10].

In vertebrates, the FGF family reflects substantial diversity, encompassing 22 FGF ligands identified in mice and humans. These FGFs can be classified into six subfamilies [11], comprising paracrine subclasses such as FGF1, FGF2, FGF3, FGF4, FGF5, FGF6, FGF7, FGF8, FGF9, FGF10, FGF16, FGF17, FGF18, FGF16, FGF20, FGF22, as well as an endocrine subclass involving FGF19, FGF21, and FGF23. The paracrine subfamily controls multiple events during embryonic development, while members of the endocrine subfamily play a significant role in regulating metabolism [12–16].

**Fig. 1:**
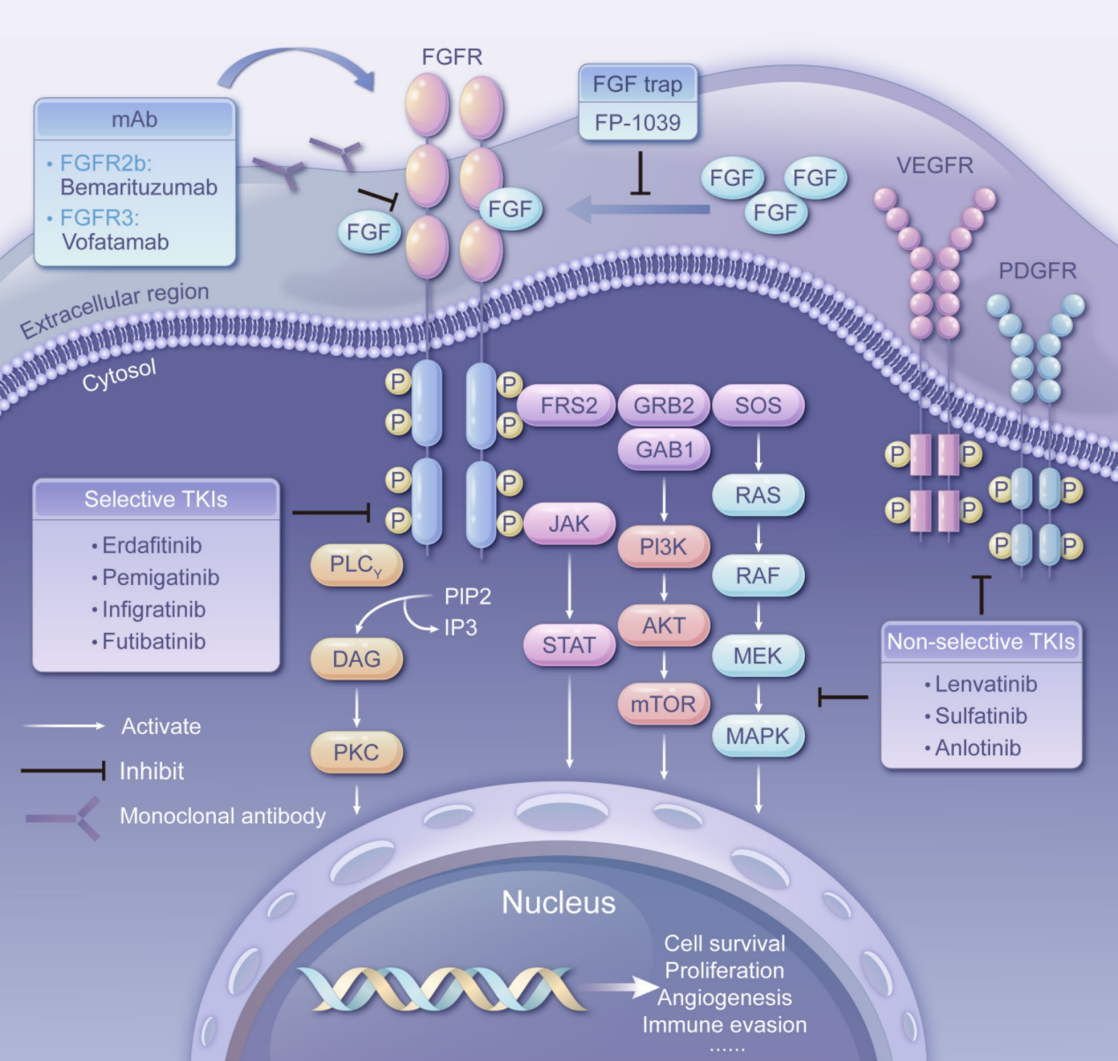
FGFR signals and inhibitors. This interaction triggers a downstream signaling network that encompasses the Ras-Raf-MAPK, PI3K-AKT, PLCγ, and STATs pathways, resulting in a range of cellular responses, including proliferation, differentiation, survival, migration, and angiogenesis

FGFR assumes the architecture of a single-pass transmembrane protein, encompassing extracellular, transmembrane, and intracellular tyrosine kinase domains [17, 18] (Fig. 1). The interaction between FGF and FGFR necessitates the presence of heparan sulfate (HS) as a co-receptor, while heparan sulfate proteoglycans (HSPG) stabilize this binding phenomenon [19, 20]. The canonical downstream signaling routes of FGF/FGFR encompass the Ras/Raf-MEK-MAPK (mitogen-activated protein kinase) pathway, phosphatidylinositol-3 kinase/protein kinase B (PI3K/AKT) pathway, PLCγ pathway, as well as signaling intermediates and transcription activators (STATs) [7, 21]. A unique docking protein, FRS2, exists in the FGFR signaling pathway. FRS2 specifically binds to members of the FGFR family through its phosphorylated tyrosine-binding structural domain, which is rare in other receptor tyrosine kinases [22]. Upon binding to the FGFR, FRS2 is phosphorylated and serves as a hub for the recruitment of downstream signaling molecules (Grb2 and Shp2), which in turn activate pathways such as the MAPK and PI3K-AKT pathways [23]. This signaling interplay is not only shaped by binding specificity, expression levels, and alternative splicing but is also subject to cross-regulation with other pathways such as BMP and Wnt [24–28]. Furthermore, post-translational modifications (including phosphorylation, glycosylation, ubiquitination) and cytoplasmic trafficking contribute substantively to the orchestration of signal specificity and strength [29–33].

In a word, the FGF/FGFR signaling pathway assumes multifarious and pivotal roles within biological systems. A comprehensive exploration of this signaling pathway promises enhanced insights into its functional intricacies and potential therapeutic applications across disease contexts.

## Prevalence and diversity of *FGFR* abnormalities in various malignancies

**Fig. 2:**
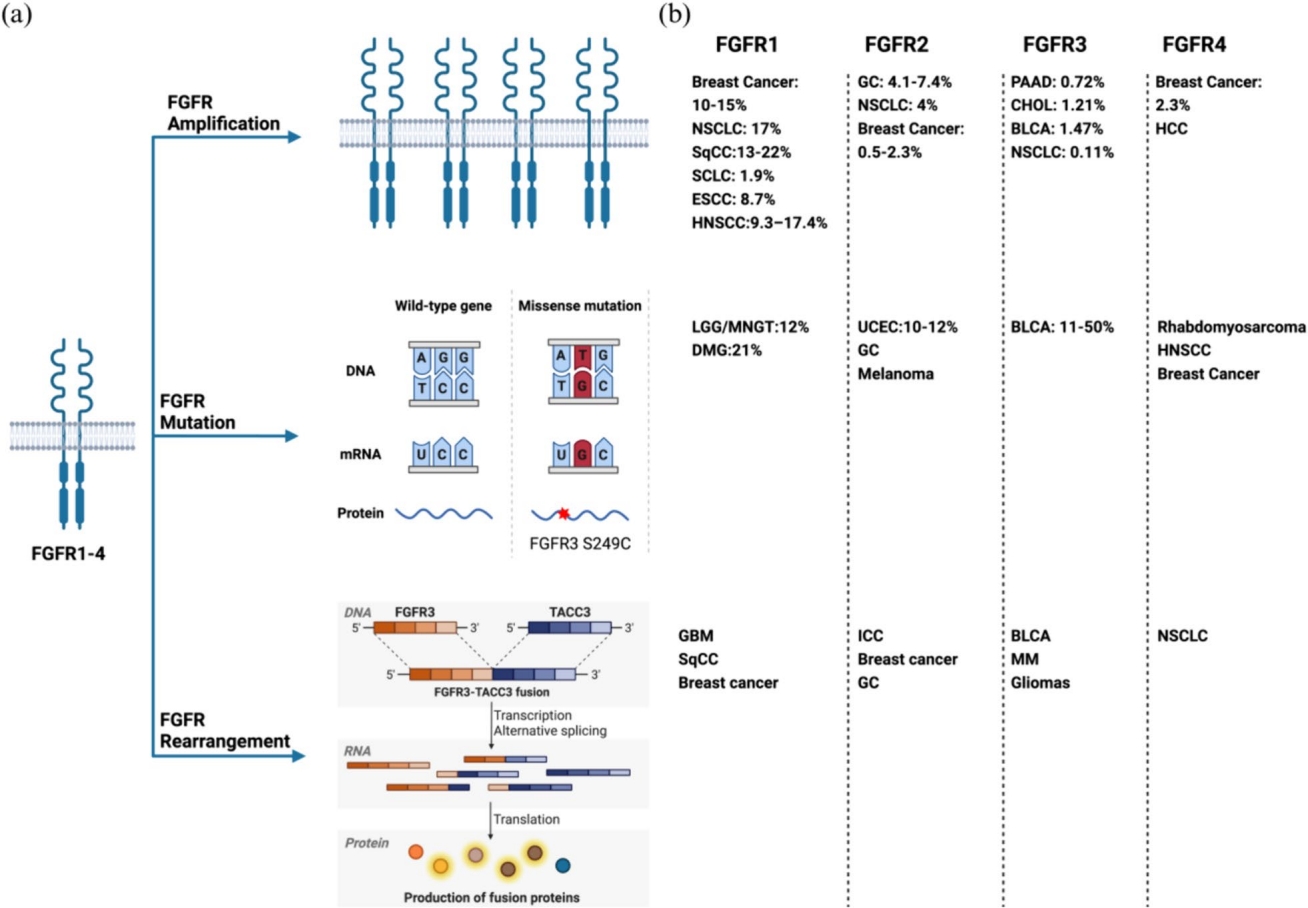
Displays a summary of FGFR alterations in cancers. **a** FGFR alterations may be categorized as amplification, point mutation, and rearrangement. The graphic illustrates the usage of FGFR3 S249C mutation and FGFR3-TACC3 for a basic mechanism explanation. **b** Presence of FGFR in various tumors. (NSCLC: non-Small cell lung carcinoma; SqCC: squamous cell cancer; SCLC: small cell lung carcinoma; ESCC: esophageal squamous cell carcinoma; HNSCC: head and neck squamous cell carcinoma; LGG: low-grade glioma; MNGT: mixedneuronal-glialtumors; DMG: diffuse midline gliomas; GBM: glioblastoma; GC: gastric cancer; UCEC: uterine corpus endometrial carcinoma; ICC: intrahepatic cholangiocarcinoma; PAAD: pancreatic cancer; CHOL: cholangiocarcinoma; BLCA: bladder cancer; MM: multiple myeloma; HCC: hepatocellular carcinoma)

Multiple sequencing investigations have shown that *FGFR* abnormalities were detected in 1.9–7.1% of tumor patients. Most of these abnormalities were gene amplifications (53.7–66%), followed by mutations (26–38.8%) and rearrangements/fusions (5.6–8%). The frequencies of aberration for *FGFR*1, *FGFR*2, *FGFR*3, and *FGFR*4 were 49–56.8%, 14.2–19%, 17.7–26% and 2.8–7%, respectively (Fig. 2). The prevalence of various tumor types was as follows: uroepithelial carcinoma (32–14.8%), colorectal carcinoma (31%), breast carcinoma (12.6–18%), gastric carcinoma (16.8–25.6%), endometrial carcinoma (13%), squamous lung carcinoma (6.8–13%), esophageal carcinoma (12.7%), ovarian carcinoma (9%), and lung adenocarcinoma (1.3%) [34–39].

### *FGFR* amplification in tumors

The phenomenon of increased copy number of the *FGFR* gene in cells is called gene amplification. A genetic sequencing of more than 4000 cancer patients showed that *FGFR* aberrations were found in 7.1% of tumor patients, most of which were gene amplifications (66% of aberrations) [40]. Carrying *FGFR* amplification is associated with adverse clinical outcomes. *FGFR*1 amplification was significantly associated with shorter overall survival (OS) (58.6 months vs. 80.0 months) in patients with squamous cell lung cancer (SqCC) [41]. *FGFR*1 amplification occurs in approximately 10% of breast cancers and functions to drive enhanced ligand-dependent signaling and suppress progesterone receptor expression, associated with poor prognosis [42]. The analysis of circulating tumor DNA (ctDNA) shows that gastric cancer patients with *FGFR*2 amplification have significantly shorter OS than those without *FGFR*2 amplification [43]. *FGFR* autophosphorylation (Tyr653/654 cyclic phosphorylation) is significantly increased in cell lines with high levels of *FGFR*2 amplification (arbitrarily defined as FISH ratio > 5), leading to *FGFR* hyperactivation [43]. Patients with high levels of *FGFR*2 amplification are thought to be likely to respond to FGFR inhibitors, but this occurs in only 5% of GC [43]. Studies have shown that *FGFR*1 is amplified in 19% of lung squamous cell carcinoma cases (14/73) and is more common in poorly differentiated tumors, suggesting a possible role in tumor aggressiveness [44]. Of these, 50% (7/14) showed high amplification (defined as real-time PCR fold greater than tenfold). And patients with high copy numbers showed better response rates to the FGFR inhibitor AZD4547 [45]. The question was raised as the research progressed: Does *FGFR* amplification have to reach a certain threshold to benefit from FGFR inhibitors?

### *FGFR* point mutation in tumors

*FGFR* mutations lead to abnormal activation of the receptor, resulting in continued activation of the FGFR signaling pathway. This sustained signaling pathway activation is a key factor in the development of many cancers, including bladder cancer, breast cancer, and non-small cell lung cancer (NSCLC) [7].

Common *FGFR*1 point mutation sites are: N546K, K656E and V561M. These mutation sites are mainly located in the kinase domain of FGFR1 and are associated with abnormal activation of FGFR1 and various diseases, including cancer. The presence of *FGFR*1 point mutations (N546K and K656E) was exclusively observed in H3K27M-mutant diffuse midline gliomas (DMG) (64/304, 21%), a subset that displayed a higher incidence in older individuals with diencephalic tumors [46]. Additionally, analogous findings have been documented for other central nervous system neoplasms, thereby establishing a correlation with heightened malignancy, diminished responsiveness to FGFR inhibitors, and occurrence of spontaneous hemorrhage [47–50].

However, in tumors with *FGFR*2 point mutations, the common sites are: S252W, N549K, E565A, K660N/K660E and V565I/V565L. The development of triple-negative breast cancer is facilitated by activating mutations in *FGFR*2-S252W, which induce epithelial-mesenchymal transition through FGFR2-STAT3 signaling [51]. The pan-cancer analysis demonstrated that uterine endometrial carcinoma (UCEC) exhibits the highest prevalence of *FGFR*2 mutations, with the most frequent mutations occurring at S252W and N549K. These mutations have been found to have an oncogenic functional impact; however, they show limited responsiveness to targeted therapy [52].

*FGFR*3 mutation sites are also different, with S249C, Y373C, G370C, R248C and V555M being the most common. The analysis of data obtained from The Cancer Genome Atlas (TCGA) demonstrated that the presence of targetable mutations in the *FGFR*3 gene was predominantly observed in bladder cancer. Specifically, the mutations S249C, Y373C, G370C, and R248C were identified as hotspot mutations, which can be effectively targeted by the FDA-approved drug erdafitinib [53]. Additionally, the occurrence of *FGFR*3 S249C mutation was less frequent in upper tract urothelial carcinoma patients compared to bladder cancer patients (37.5% vs. 59.3%) [54].

*FGFR*4 mutation sites with high incidence are as follows: V550L/V550M/V550E/N535D/N535K and G388R. Genomic analysis of rhabdomyosarcoma reveals high prevalence of FGFR4 overexpression and mutations in tumor tissues as a result of PAX-FOXO1 oncogene transcription [55].

### *FGFR* gene rearrangement and fusion in tumors

Gene rearrangement is the process by which genes are rearranged on chromosomes. This rearrangement can be caused by chromosome breakage, transposition, inversion, or translocation [56]. These changes can result in the loss of gene function, the production of abnormal gene proteins, or the activation of previously dormant oncogenes [57].

Gene fusions involving *FGFR*1, and various partner genes have been identified in diverse tumor types. The *FGFR*1-*TACC*1 fusion is detected in glioblastoma and SqCC. This fusion is associated with the hyperactivation of FGFR1, which promotes cellular proliferation and inhibits apoptosis [58]. Additionally, it might augment FGFR1 signaling either by intensifying its tyrosine kinase activity or by modulating its intracellular positioning [59]. *BCR-FGFR*1, *FGFR*1OP-*RET*, and *FGFR*1OP-*FGFR*1 Fusions, which are identified in myeloproliferative disorders, have been associated with the initiation and advancement of the disease [60–62]. They might bolster cell survival by facilitating cell cycle progression and suppressing apoptosis.

Gene fusions between *FGFR*2 and various genes are commonly identified in cholangiocarcinoma (CCA), playing a crucial role in tumorigenesis and tumor progression [63]. The presence of the *FGFR*2-*BICC*1 fusion is associated with the aggressive and malignant characteristics of cholangiocarcinoma [63]. The *FGFR*2 fusion necessitates the involvement of the downstream effector Mek1/2, indicating the potential clinical efficacy of dual blockade targeting FGFR2 and MEK1/2 in patients with intrahepatic cholangiocarcinoma (ICC) [63]. *FGFR*2- *PPHLN*1, *AHCYL1*, and *TACC*3 fusions might potentially result in continuous activation of *FGFR*2, which in turn promotes the initiation and progression of CCA [64]. A noteworthy finding is that 90% of patients diagnosed with ICC and having *KRAS* mutations also showed positive findings for *FGFR*2 fusions, suggesting a potential collaborative role in promoting the progression of malignant tumors [65]. Consequently, it is hypothesized that the occurrence of *FGFR*2 fusion in ICC might serve as an initial genetic occurrence that fosters the initiation and progression of tumorigenesis [66].

Gene fusions of *FGFR*3 with multiple partners are prevalently reported in bladder cancer, gliomas, and multiple myeloma, playing a central role in tumorigenesis and progression [67–69]. The *FGFR*3-*TACC*3 fusion protein is shown to be localized to mitotic spindle poles, according to research results, possesses inherent kinase activity, elicits abnormalities in mitosis and chromosome segregation, and instigates aneuploidy, ultimately leading to the development of tumorigenicity [70]. The fusion alteration in female bladder cancer patients is regarded as a significant risk factor [71]. The fusion of *FGFR*3-*TACC*3 has recently been identified as a relatively uncommon yet potentially significant mechanism of resistance to EGFR inhibitors, specifically osimertinib, in cases of lung cancer [72]. The t(4;14) transforming events lead to activation of FGFR3 in myeloma, and such patients have poor survival and response to chemotherapy [69]. Efficacy is currently being evaluated in a combination trial of erdafitinib + dexamethasone in these patients (NCT02952573). Bladder cancer is associated with *FGFR*3-*BAIAP2L*1 fusions, which are known to contribute to the aggressive nature and malignancy of the disease [73]. They might boost FGFR3 signaling by increasing its stability or modifying its intracellular positioning.

Gene fusions of *FGFR*4 are relatively rare, and one fusion mutation, *FGFR*4-RAPGEFL1, has been identified in NSCLC. Its function remains unclear and requires further investigation [74].

## Detection of FGFR alterations in tumors

### Fluorescence in situ hybridization

Fluorescence in situ hybridization (FISH) employs fluorescently labeled probes to hybridize with target DNA sequences. This allows researchers to directly visualize the location and quantity of chromosomes or genes within cellular or tissue sections. In the realm of *FGFR* gene studies, FISH has been established as a method of high sensitivity and specificity, adept at identifying amplifications, mutations, and fusions associated with *FGFR* genes [75]. The excellent spatial resolution of FISH compared to other assays makes FISH particularly effective at mapping heterogeneous changes in the FGFR gene at the single-cell level [76]. As with the Next-Generation Sequencing (NGS) assay, the latest National Comprehensive Cancer Network (NCCN) guidelines recommend it as a test for *FGFR*2 fusion/other *FGFR* aberrations in cholangiocarcinoma [77].

### Reverse transcription-polymerase chain reaction

Reverse transcription-polymerase chain reaction (RT-PCR) is an experimental method by first converting RNA template into cDNA, and then using PCR technology for DNA amplification. With repeated cycles, RT-PCR can significantly enhance the signal of the target sequence, which allows RT-PCR to detect and quantify specific RNA molecules starting from extremely small amounts of RNA [78, 79]. Both NCCN and European Society for Medical Oncology (ESMO) guidelines recommend the detection of *FGFR*2/3 mutations and fusions in bladder cancer, and the FDA has also approved the therascreen® FGFR RGQ RT-PCR kit as a PCR-based companion diagnostic kit [80, 81]. The combination of peptide nucleic acid mediated RT-PCR clamping has been shown to be sensitive enough to detect FGFR3 mutations in bladder cancer from urine sediments, clearly distinguishing mutant DNA from wild-type DNA at concentrations greater than 1% [82]. In contrast to alternative methodologies, such as sequencing, RT-PCR can deliver results in a significantly shorter timeframe, often within hours. It has been instrumental in identifying *FGFR* fusions, particularly evident in malignancies like cholangiocarcinoma [83].

### Next-generation sequencing

NGS is a revolutionary DNA sequencing technology. The basic principle is to break a DNA sample into millions of small fragments, and then these fragments are amplified, sequenced, and analyzed through different methods, which can simultaneously sequence millions to billions of DNA fragments [84]. Targeted NGS panels, focusing on specific genes or gene regions, have been developed for comprehensive *FGFR* profiling [85]. These panels can detect point mutations, copy number variations, and gene fusions with high sensitivity and specificity. Several studies employing NGS have reported a diverse spectrum of *FGFR* alterations across different tumor types [40, 75]. Based on the advantages of NGS testing and past practical experience, the latest NCCN and ESMO guidelines recommend it as a detection method for *FGFR*2 fusion/other *FGFR* aberrations in cholangiocarcinoma [77, 86]. ESMO guidelines emphasize that detection involving FGFR2 is best performed at the RNA level to help identify fusion transcripts with unknown fusion partners [86]. In bladder cancer, ESMO guidelines recommend NGS for FGFR2/3 mutation and fusion detection [80].

### Hybridization-capture

Hybridization-capture is a genomic technique that uses specialized probes to capture and enhance target DNA sequences, allowing for rapid and precise sequencing analysis [87]. Hybridization-capture enables a thorough examination of the genetic makeup of tumors, allowing for the detection of hitherto unidentified FGFR fusions [88]. By using this technique in NGS, it becomes possible to analyze a substantial quantity of samples concurrently, therefore offering a comprehensive depiction of FGFR abnormalities among the whole population [89]. Hybridization capture may be used to discover FGFR fusions in patients who do not respond to conventional treatments. These fusions can serve as acquired resistance mechanisms and studying them can assist improve the prognosis of the patients [89].

### Liquid biopsy

Liquid biopsy is an innovative diagnostic technique that analyzes biomarkers in body fluids like blood, saliva, or urine to detect and monitor diseases, particularly cancers. This technology captures circulating tumor cells, ctDNA, microRNAs, and other biomolecules, offering a non-invasive alternative to traditional tissue biopsies [90, 91]. Research has underscored the potential of liquid biopsy in identifying *FGFR* alterations. For instance, *FGFR* mutations and fusions, once identified in tissue samples, have been detected in ctDNA with a high degree of concordance [92]. NCCN guidelines also recognize that the use of cell free DNA (cfDNA) testing can detect some *FGFR*2 fusion breakpoints in cholangiocarcinoma, but the sensitivity is lower than that of tumor tissue testing [77]. Moreover, liquid biopsies facilitate continuous monitoring, capturing the evolving profile of *FGFR* alterations during treatment, which could signal treatment resistance or disease progression [93].

### Targeting FGFR in the clinic

**Fig. 3:**
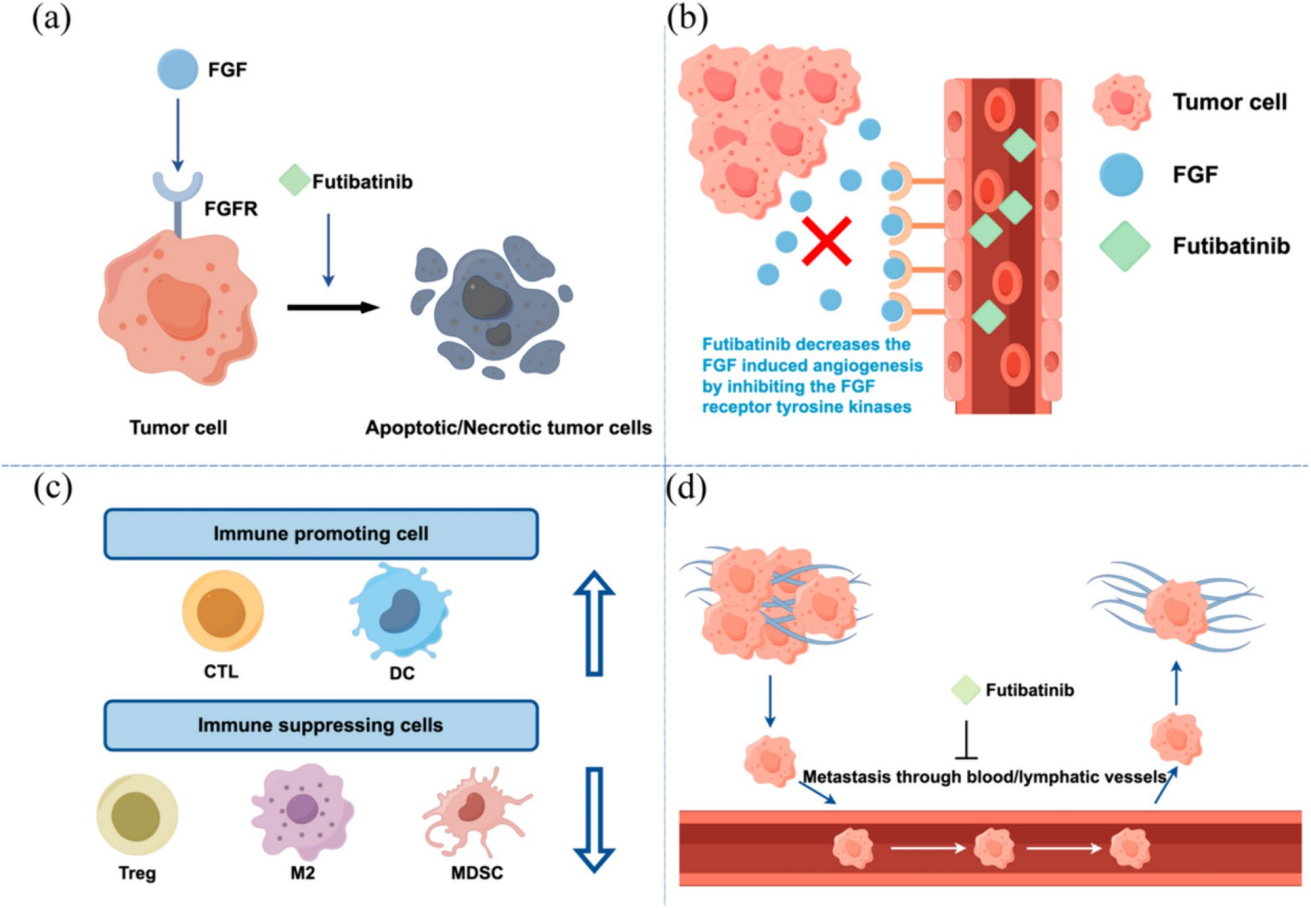
Take Futibatinib as an example, showing the functions of FGFR inhibitors. **a** Cell apoptosis. **b** Antiangiogenic impact. **c** Regulation of the immunological microenvironment. **d** Anti-metastatic impact

The contribution of aberrant FGFR signaling to tumorigenesis has led to the development of multiple therapies targeting the FGFR pathway (Fig. 3), many of which have shown promise in phase II and III clinical studies in various tumor types with FGFR abnormalities (Tables 1 and 2). We focused on selective FGFRs inhibitors with Phase II/III clinical trial results and representative multi-target kinase inhibitors.

**Table 1:** Summary of FGFR inhibitors currently being investigated in clinical trials

Inhibitor (drug name)	Target	Previous treatment	Clinical trial number	Phase	Cancer type	Results	Major adverse events
*Selective inhibitors*							
Infigratinib	FGFR1-3	Received ≥ 1 prior gemcitabine-containing regimens	NCT02150967	II	Advanced/metastatic CCA	ORR: 14.8% DCR: 75.4% Median PFS: 5.8 months	TRAEs: Hyperphosphatemia: 72.1% (all grades) Fatigue: 36.1% Stomatitis: 29.5% Alopecia: 26.2%
Pemigatinib	FGFR1-3	Received ≥ 1 prior systemic regimens	NCT02924376	II	Advanced/metastatic CCA	ORR:35.5%	TRAEs: Hyperphosphataemia: 60% Arthralgia: 6% Stomatitis: 5% Hyponatraemia: 5% Abdominal pain: 5% (including serious: 5%) Fatigue: 5% Pyrexia (serious): 5% Cholangitis (serious): 3% Pleural effusion (serious): 3%
Pemigatinib	FGFR1-3	Received ≥ 1 prior systemic regimens	NCT03011372	II	Myeloid or lymphoid tumors	CR rates: Investigator (64.5%), CRC (77.4%)	TRAEs: Hyperphosphatemia: 68% Alopecia: 59% Diarrhea: 50% Stomatitis: 44% (grade ≥ 3: 12%) Anemia (grade ≥ 3): 18%
Erdafitinib	FGFR1-4	Received ≥ 1 prior systemic regimens	NCT02365597	II	UC	Response: 40% (CR: 3%, PR: 37%) PFS: 5.5 months OS: 13.8 months	TRAEs (grade 3 or higher): 46% of patients Discontinuation due to adverse events: 13%
Erdafitinib	FGFR1-4	Received ≥ 1 prior systemic regimens	NCT03390504	III	Advanced/metastatic UC	Median OS: 12.7 months (erda) versus 2.7 months (chemo) Risk reduction of death: 36% Median PFS: 5.6 months (erda) versus 2.7 months (chemo) ORR: 46% (erda) versus 12% (chemo)	TRAEs: 13% (erda), 24% (chemo) Grade 3/4 TRAEs: 46% (both erda and chemo) TRAEs leading to death: 1 (erda), 6 (chemo) Dose reductions: 66% (erda), 21% (chemo) Discontinuation due to TRAEs: 8% (erda), 13% (chemo) Central serous retinopathy: 17% (erda), Grade 1–2 (20 patients)
Erdafitinib	FGFR1-4	Progressed after ≥ 1 prior systemic regimens (including platinum or gemcitabine based therapies in many cases)	NCT02699606	II	Advanced CCA	ORR: 50% (overall), 60% (in FGFR2 + patients) DCR: 83.3% (overall), 100% (in FGFR2 + patients) Median DOR: 6.83 months Median PFS: 5.59 months (overall), 12.35 months (in FGFR2 + patients)	Common TEAEs (> 30%): hyperphosphatemia, dry mouth, stomatitis, and dry skin ≥ Grade 3 adverse events (AEs): 9 patients (7 drug-related), including 1 death (not drug-related) Treatment adjustments due to TEAEs: 1 discontinuation, 6 dose reductions
Erdafitinib	FGFR1-4	N/A	NCT03210714	II	Glioma or glioneuronal tumor (low and high grade), Neuroblastoma, Osteosarcoma, Rhabdomyosarcoma	2 patients (10%) had a PR Stable disease was observed in 6 additional patients with gliomas with a median duration of 6.5 cycles The 6-month PFS: 45% The 6-month OS: 89.7%	TRAEs: Hyperphosphatemia Nail changes or infections Vision changes: Grade 1 Spinal cord compression: 1 case (Grade 3) Intracranial hemorrhage: 1 case (Grade 4)
Futibatinib	FGFR1-4	Received ≥ 1 prior systemic regimens	NCT02052778	I/II	Unresectable/metastatic ICC	ORR: 42% Median PFS: 9.0 months Median OS: 21.7 months	Common grade 3 TRAEs: Hyperphosphatemia: 30% Increased aspartate aminotransferase level: 7% Stomatitis: 6% Fatigue: 6% Permanent discontinuation due to adverse events: 2% of patients Treatment-related deaths: None
Futibatinib	FGFR1-4	received ≥ 2 prior systemic regimens	NCT04189445	II	GC/GEJC	ORR: 17.9% DCR: 50.0% Median time to response: 1.6 months Median DOR: 3.9 months Median PFS: 2.8 months Median OS: 5.7 months	Common TRAEs: Hyperphosphatemia: 89.3% (all grade 1 or 2) Decreased appetite: 32.1% Increased aspartate aminotransferase: 21.4% Increased alanine aminotransferase: 17.9% Diarrhea: 17.9% Nausea: 14.3% Fatigue: 14.3% Grade 3 TRAEs: Anemia: n = 2 Decreased appetite: n = 3 Grade 4 TRAE: Decreased neutrophil count: n = 1 No TRAEs resulted in death
AZD4547	FGFR1-3	Received ≥ 1 prior gemcitabine-containing regimens	S1400D (Part of Lung-MAP)	II	NSCLC	Median PFS: 2.7 months Median OS: 7.5 months	Grade 3 TRAEs: occurred in 6 patients Grade 4 TRAEs: sepsis in 1 patient Other TRAEs: not detailed
AZD4547	FGFR1-3	N/A	NCT02465060	II	Various, including breast (33.3%), urothelial (12.5%), and cervical cancer (10.4%)	Stable disease was observed in 37.5% Median PFS: 3.4 months 6-month PFS rate: 15% For patients with FGFR fusions, the response rate: 22% 6-month PFS rate: 56%	Grade 3 TRAEs were consistent with those reported in previous clinical trials (specific events not detailed)
HMPL-453	FGFR1, 2, 3	Received ≥ 1 prior systemic regimens	NCT04353375	II	CCA/ICC	DCR: 86.4% ORR: 31.8% In cohort 1, Median PFS: 5.7 months In cohort 2, the confirmed ORR was 50% and DCR was 90%	Common TRAEs: Diarrhea: 56% Dry mouth: 44% Increased blood phosphorus: 44% Grade ≥ 3 TRAEs (each occurring in 8% of patients): Decreased neutrophil count Nail toxicity Palmar-plantar erythrodysesthesia syndrome
RLY-4008	FGFR2	Received ≥ 1 prior systemic regimens	NCT04526106	I/II	CCA	ORR: 63.2% DCR: 94.7%	Most common TRAEs: Stomatitis: 48% Palmar-Plantar Erythrodysesthesia (PPE): 46% Dry mouth: 31% Grade 4/5 TRAEs: None reported
Rogaratinib	FGFR1-4	Received ≥ 1 prior platinum-containing regimens	NCT03410693	II/III	Advanced/Metastatic UC	ORR: 20.7% (rogaratinib), 19.3% (chemo) Median OS: 8.3 months (rogaratinib), 9.8 months (chemo)	Grade 3 TRAEs: Rogaratinib: 43.0% Chemotherapy: 39.0% Grade 4 TRAEs: Rogaratinib: 4.7% Chemotherapy: 18.3% Rogaratinib-related deaths: None
FGF401	FGFR4		NCT02325739	I/II	HCC	4 Patients experienced objective responses (4 PR)	Frequent TRAEs: Diarrhea: 73.8% Increased AST (aspartate aminotransferase): 47.5% Increased ALT (alanine aminotransferase): 43.8% Grade 3 dose-limiting toxicities: Increase in transaminases: n = 4 Increase in blood bilirubin: n = 2
*Non-selective inhibitors*							
Lenvatinib	VEGFR, FGFR, PDGFR	Received ≥ 1 prior systemic regimens	NCT03713593	III	Advanced HCC	Median OS: 21.2 months (Lenva + Pembro) versus 19.0 months (Lenva alone) ORR: 26.1% (Lenva + Pembro) versus 17.5% (Lenva alone)	Grade 3–5 TRAEs: Lenva + Pembro arm: 62.5% Lenva arm: 57.5% Grade 5 TRAEs: Lenva + Pembro arm: 1.0% Lenva arm: 0.8%
Lenvatinib + Pembrolizumab	VEGFR, FGFR, PDGFR, PD-1	No previous systemic therapy	NCT02811861	III	Advanced RCC	PFS (PFS): Lenvatinib + Pembrolizumab: 23.9 months Hazard Ratio (HR) for disease progression or death: 0.39 HR for death: 0.66	Grade 3–5 TRAEs: 62.5% versus 57.5% (lenva + pembro vs. lenva arm) Grade 5 TRAEs: 1.0% versus 0.8% (lenva + pembro vs. lenva arm)
Lenvatinib + Everolimus	VEGFR, FGFR, PDGFR, mTOR	No previous systemic therapy	NCT02811861	III	Advanced RCC	PFS: 14.7 months (Lenvatinib + Everolimus) versus 9.2 months (Sunitinib) HR for disease progression or death: 0.65 HR for death: 1.15	Grade 3–4 TRAEs > 10% of patients: Hypertension Diarrhea Elevated lipase levels Grade 3–4 TRAEs in patients on Lenvatinib + Everolimus treatment: 83.1%
Lenvatinib + Pembrolizumab	VEGFR, FGFR, PDGFR, PD-1	Received ≥ 1 prior platinum-containing regimens	NCT03517449	III	Advanced Endometrial Cancer	OS (pMMR): 17.4 months (Lenvatinib + Pembrolizumab) vs. 12.0 months (Chemo) Overall OS: 18.3 months versus 11.4 months PFS (pMMR): 6.6 months (Lenvatinib + Pembrolizumab) versus 3.8 months (Chemo) Overall PFS: 7.2 months versus 3.8 months	Grade 3–4 TRAEs: Lenvatinib + Pembrolizumab: 88.9% Chemotherapy: 72.7%
Surufatinib	VEGF, FGFR, CSF1R	Received ≤ 2 prior systemic regimens	NCT02588170	III	Advanced, well differentiated extrapancreatic NETs	PFS: 9.2 months (surufatinib) versus 3.8 months (placebo)	TRAEs: Hypertension: Surufatinib group: 36% Placebo group: 13% Proteinuria: Surufatinib group: 19% Placebo group: 0% Serious TRAEs: Surufatinib group: 25% Placebo group: 13% Treatment-related deaths: Surufatinib group: 3 patients Placebo group: 1 patient
Nintedanib + Docetaxel	VEGFR, PDGFR, FGFR	Received ≥ 1 prior platinum-containing regimen	NCT00805194	III	NSCLC	PFS: Docetaxel + Nintedanib: 3.4 months Docetaxel + Placebo: 2.7 months OS for Adenocarcinoma within 9 months: Docetaxel + Nintedanib: 10.9 months Docetaxel + Placebo: 7.9 months OS for All Adenocarcinoma Patients: Docetaxel + Nintedanib: 12.6 months Docetaxel + Placebo: 10.3 months OS for Total Study Population: Docetaxel + Nintedanib: 10.1 months Docetaxel + Placebo: 9.1 months	TRAEs: Docetaxel + Nintedanib versus Docetaxel + Placebo Diarrhoea: 6.6% versus 2.6% Increases in ALT: 7.8% versus 0.9% Increases in AST: 3.4% versus 0.5% Deaths (not disease-related): 35 versus 25
Nintedanib	VEGFR, PDGFR, FGFR	No previous systemic regimens	NCT01907100	II/III	Unresectable epithelioid malignant pleural mesothelioma	PFS: Nintedanib: 6.8 months versus Placebo: 7.0 months	TRAEs: Neutropenia: Nintedanib: 32% Placebo: 24% Serious Adverse Events: Nintedanib: 44% Placebo: 39% Pulmonary Embolism: Nintedanib: 6% Placebo: 3%
Anlotinib	VEGFR, PDGFR, FGFR	Received ≥ 3 prior systemic regimens	NCT02388919	II/III	Advanced NSCLC	OS: 9.6 months (Anlotinib) versus 6.3 months (Placebo) PFS: 5.4 months (Anlotinib) versus 1.4 months (Placebo)	Grade 3–4 TRAEs: Hypertension Hyponatremia
Lucitanib	VEGFR, PDGFR, FGFR	Received ≤ 2 prior systemic regimens	N/A	II	HR+/HER2− metastatic breast cancer	Cohort 1 ORR: 19% Cohort 2 ORR: 0% Cohort 3 ORR: 15%	TRAEs: Hypertension: 87% Hypothyroidism: 45% Nausea: 33% Proteinuria: 32%
Derazantinib	VEGFR, PDGFR, FGFR, RET, KIT	Received ≥ 1 prior systemic regimens	NCT03230318	II	FGFR2M/A + ICC	Treatment Response: PR: 2 patients (8.7%) SD: 15 patients (65.2%) DCR: 73.9% (95% CI: 51.6–89.8) PFS: Median PFS: 7.3 months 3-month Progression-Free Probability: 76.3% 6-month Progression-Free Probability: 50.3%	N/A
Tinengotinib	Aurora, FGFR, VEGFRs, JAK CSF1R	Exhausted standard treatment options	NCT04919642	II	CCA	Cohort A2: PR Achieved: 2/6 pts (33%) with tumor reductions of 34% and 54% DCR by FGFR Status: FGFR2 fusion/rearrangement: 90% (9/10) FGFR primary mutation: 100% (1/1) FGFRwt: 71% (5/7) Duration: One PR and 5 SDs lasted > 16 weeks	TRAEs: Total: 20/25 pts (80%) G1-2 AEs: 7/25 pts (28%) G3 AEs: 12/25 pts (48%) G4 AE: 1/25 pts (4%) Frequent G3/4 TRAEs: Hypertension: 6/25 pts (24%) G3 Fatigue: 2/25 pts (8%) G3 Neutrophil Count Decrease: 2/25 pts (8%)
*Monoclonal antibody*							
Vofatamab	FGFR3 mutations or fusions	Received ≥ 1 prior systemic regimens	NCT03123055	I/II	Metastatic UC	WT Patient Responses: Total Responses: 6/20 (30%) Confirmed: 4 Unconfirmed: 1 iRECIST Response: 1 Treatment Continuation: 13/20 (65%) remain on treatment after 4 + months Median Cycles: 8 (Range: 1–13) PFS: At 5 + months, median PFS not reached	TEAE (> 20% of patients): Nausea Anemia Diarrhea Fatigue
Bemarituzumab	FGFR2b	N/A	NCT03694522	II	GC/GEJC	PFS: Bemarituzumab: 9.5 months Placebo: 7.4 months HR: 0.68	Bemarituzumab versus Placebo Group (Grade 3–4 TRAEs): Decreased Neutrophil Count: 30% (23/76) versus 35% (27/77) Cornea Disorder: 24% (18/76) versus 0% Neutropenia: 13% (10/76) versus 9% (7/77) Stomatitis: 9% (7/76) versus 1% (1/77) Anaemia: 8% (6/76) versus 13% (10/77) Treatment-Related Deaths (Bemarituzumab): Total: 3 patients (2-Sepsis, 1-Pneumonia)

**Table 2:** Drugs undergoing phase III clinical trials and their structural formula

Inhibitor (drug number)	Structural formula	Target	Key inclusion criteria	Study design	Primary endpoint	Clinical trial number	Cancer type	Estimated enrollment
Infigratinib	(See Fig. 5)	FGFR1, 2, and 3	FGFR2 Fusion/rearrangement	RCT phase III	PFS	NCT03773302	Advanced CCA	Approximately 300
Pemigatinib	(See Fig. 5)	FGFR1, 2, and 3	FGFR2 Fusion/rearrangement	RCT phase III	PFS	NCT03656536	Advanced CCA	–
Futibatinib	(See Fig. 5)	FGFR1-4	FGFR2 Rearrangements	RCT phase III	PFS	NCT04093362	Metastatic/unresectable ICC	Approximately 216
Lenvatinib + Pembrolizumab		VEGFR FGFR PDGFR	Not specified	RCT phase III	PFS and OS	NCT04949256	Metastatic ESCC	Approximately 850
			Not HER2-positive	RCT phase III	PFS and OS	NCT04662710	Advanced/metastatic AEG	–
Anlotinib		VEGFR FGFR PDGFR	RAS/BRAF WT	RCT phase III	PFS	NCT04854668	Unresectable metastatic Colorectal cancer	698
Bemarituzumab	–	FGFR2b	FGFR2b Protein overexpression	RCT phase III	OS	NCT03343301	Advanced/Metastatic AEG	–
Bemarituzumab + Nivolumab + mFOLFOX6	–	FGFR2b	FGFR2b Protein overexpression	Ib RCT phase III	OS	NCT05111626	Advanced/Metastatic AEG and GC	Approximately 528

### Listed drugs

#### Infigratinib

**Fig. 4:**
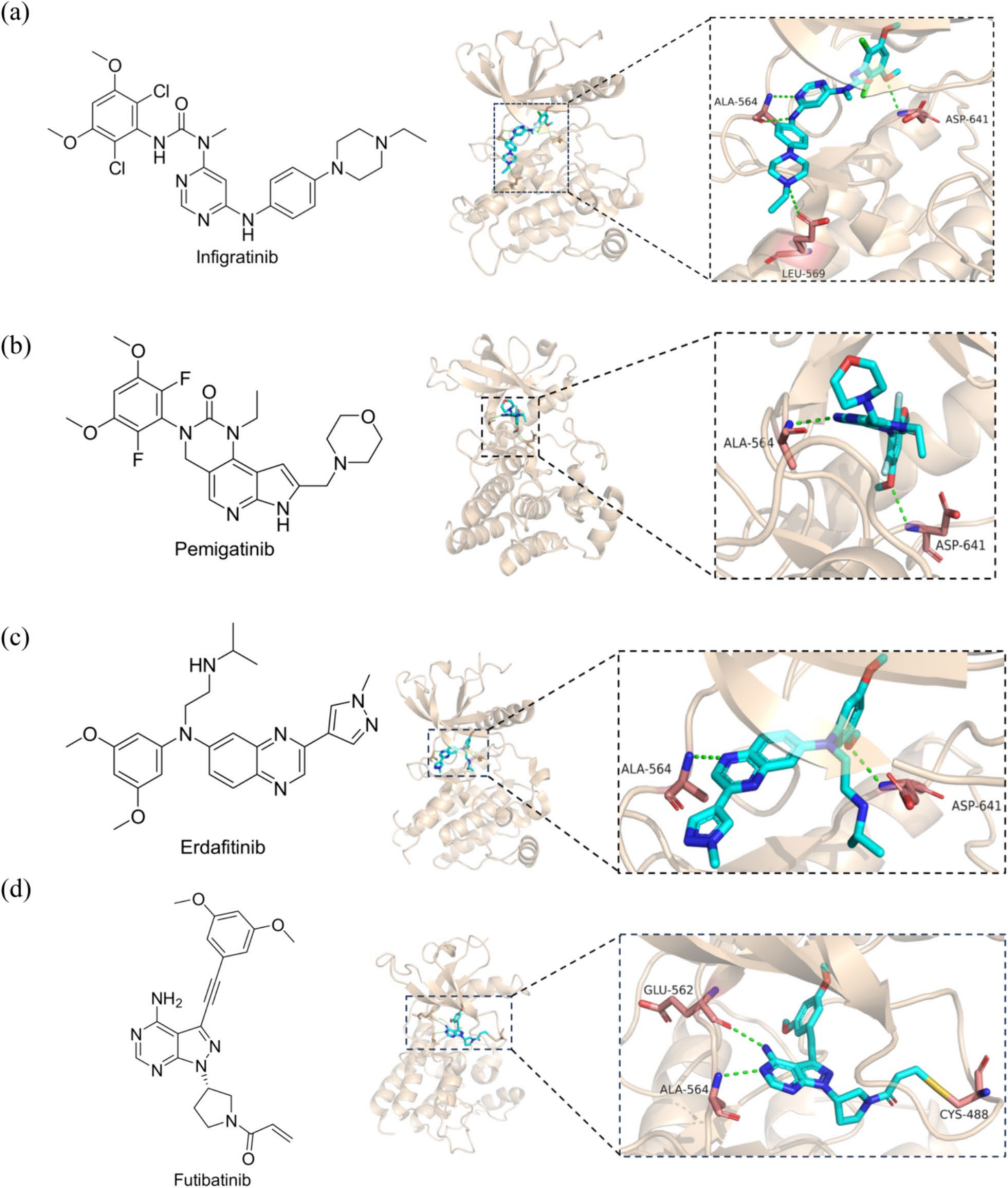
**a** Crystal structures of the compound Infigratinib and the compound Infigratinib in FGFR1 (PDB ID 3TT0). **b** Crystal structures of the compound Pemigatinib and the compound Pemigatinib in FGFR1 (PDB ID 7WCL). **c** Crystal structures of the compound Erdafitinib and the compound Erdafitinib in FGFR1 (PDB ID 5EW8). **d** Crystal structures of the compound Futibatinib and the compound Futibatinib in FGFR1 (PDB ID 6MZW)

Infigratinib is a potent and selective inhibitor of FGFR1, 2 and 3 that has demonstrated significant anti-tumor efficacy in preclinical studies [94–98] (Fig. 4a). The results of a phase II study indicate that infigratinib exhibits significant clinical activity in patients with CCA who have previously received gemcitabine treatment containing an *FGFR*2 fusion. The overall response rate (OR) was found to be 14.8%, and the disease control rate was 75.4% [99]. Following a median follow-up duration of 10.6 months, the ORR was determined to be 23.1% (25 out of 108 patients), with a patient demonstrating a complete response (CR) [100]. In particular, several phase III trials are underway evaluating infigratinib versus standard of care as a first-line treatment in patients with CCA and infigratinib versus placebo in patients with urothelial carcinoma [101, 102]. Another phase II study demonstrated restricted effectiveness of infigratinib monotherapy in patients with recurrent gliomas and various *FGFR* gene alterations. However, patients with *FGFR*1 or *FGFR*3 point mutations or *FGFR*3-*TACC*3 fusions exhibited enduring disease control exceeding 1 year [103]. Intriguingly, infigratinib appears to possess therapeutic potential in the treatment of tumour-induced osteochondrosis (TIO), an uncommon paraneoplastic syndrome [104]. However, in October 2022, Helsinn Group announced the withdrawal of infigratinib's application for listing in the United States based on business plan considerations.

#### Pemigatinib

Pemigatinib, an inhibitor targeting FGFR 1, 2, and 3, effectively inhibits the growth of FGFR-abnormalized xenografts [105–107] (Fig. 4b). In a phase II clinical study, it was observed that among patients with CCA who had previously undergone therapy and experienced of *FGFR*2 fusions or rearrangements, the administration of 13.5 mg of oral pemigatinib once daily resulted in an ORR of 35.5% with 38 out of 107 patients achieving a response, including 3 CR and 35 partial responses [108]. Furthermore, post hoc analyses of the study revealed that the median Progression-Free Survival (PFS) for patients with *FGFR*2 fusion/rearrangement (n = 65) who received second line pemigatinib was 7.0 months [109]. Another similar study found that 15 of 30 patients with *FGFR*2 fusions or rearrangements who were evaluated for efficacy achieved a partial response (ORR, 50.0%) [110]. Considering these findings, a randomized controlled phase III study (FIGHT-302) is currently underway to assess and compare the effectiveness and safety of first line pemigatinib in contrast to gemcitabine plus cisplatin in the management of patients diagnosed with advanced CCA exhibiting *FGFR*2 rearrangements [111]. In the cohort of patients with relapsed or refractory myeloid or lymphoid neoplasms harboring recombinant *FGFR*1 genes and treated with pemigatinib, the evaluation of efficacy was conducted on a total of 33 patients. The rates of complete cytogenetic response were in rates of 72.7% and 75.8%, respectively. The median duration of CR has not yet been determined [112]. Furthermore, Pemigatinib has demonstrated favorable clinical efficacy and a satisfactory safety profile in the treatment of gliomas, gynecological tumors, and pancreatic cancer [113].

#### Erdafitinib

Erdafitinib exhibits potent inhibitory activity against FGFR1-4 and shows robust antitumor effects in preclinical studies [114, 115] (Fig. 4c). In a phase II clinical trial, the objective response rate (ORR) of individuals diagnosed with locally advanced, unresectable, metastatic urothelial carcinoma (mUC) and treated with erdafitinib was found to be 40% [116]. The duration of the curative effect was observed for a period of 24.0 months. Out of a total of 101 patients, the researchers determined that the ORR for those who received the erdafitinib regimen was 40% (40%; 95% CI 30–49) [117]. The THOR 3 study showed that in patients with mUC with FGFR alterations previously treated with immunotherapy, erdafitinib significantly improved median PFS (5.6 vs. 2.7 months) and ORR (46% vs. 12%) compared with chemotherapy [118]. Data from the THOR 2 study showed a favorable and durable response to erdafitinib in patients with high-risk, BCG-nonresponsive non-muscle invasive bladder cancer (NMIBC) and FGFR genetic alterations, with 16 patients treated for a median of 6.7 months [119]. Additionally, the cohort revealed that erdafitinib did not outperform pembrolizumab in patients with *FGFR*-altered, anti-PD-(L)1-naive mUC, achieving median OS times of 10.9 and 11.1 months, respectively [120]. The RAGNAR study confirmed the antitumor effect of erdafitinib in patients with tumors with multiple *FGFR* mutations. Independent evaluations showed an ORR of 30% and a sustained response period of 6.9 months, with an ORR of 56% for pancreatic cancer (18 enrolled) and 52% for CCA(31 enrolled) [121]. TAR-210, a novel intravesical delivery system for erdafitinib, also demonstrated positive clinical activity in patients with high- and intermediate-risk NMIBC with *FGFR* alterations, with 13 (87%) of the 315 patients in the cohort with available response evaluators. Achieve complete remission [122]. In addition, PR and SD can be achieved in more than 50% of Asian patients with advanced CCA and pediatric central malignancies with FGFR alterations treated with Erdafitinib [123, 124].

#### Futibatinib

Futibatinib is an irreversible pan-FGFR inhibitor that has shown potent activity in preclinical studies against tumors that are resistant to FGFR inhibitors [125–127] (Fig. 4d). In patients with previously treated *FGFR*2 fusion- or rearrangement-positiven ICC, the use of futibatinib provided a significant clinical benefit, with a total of 43 of 103 patients (42%; 95% confidence interval 32–52) responding, with a median PFS of 9.0 months and OS of 21.7 months [128]. A Phase III trial is currently underway to assess the effectiveness and safety of futibatinib in comparison to gemcitabine-cisplatin chemotherapy as the initial therapeutic approach for individuals with advanced, metastatic, or recurrent unresectable ICC carrying a *FGFR*2 gene rearrangement [129]. In another Phase II study, TAS-120 demonstrated comparable anti-tumor activity in patients with advanced or metastatic gastric and gastroesophageal cancer harboring *FGFR*2 amplification, with median PFS and OS estimates of 2.8 months and 5.7 months [130].

As mentioned above, there are currently four FGFR inhibitors approved for marketing by the FDA, with infigratinib having been removed from the market due to commercial reasons (Table 3). In 04/2019 Erdafitinib was approved by the FDA for the treatment of uroepithelial carcinoma at a daily oral dose of 8–9 mg. Pemigatinib and Futibatinib were also approved by the FDA for the treatment of CCA in 04/2020 and 09/2022 at a daily oral dose of 13.5 mg and 20 mg, respectively. Despite their limitations, the four approved FGFR inhibitors offer relief to FGFR-altered patients who have failed other treatments, prolonged survival and bringing hope to these patients.

**Table 3:** Four FGFR inhibitors approved for marketing by the FDA

Inhibitor (drug name)	Target	Indication	FDA approval date	Dosing schedule	Efficacy results	sAE	AE	Laboratory abnormalities
Futibatinib	FGFR2 gene fusions or rearrangements	Unresectable advanced or metastatic intrahepatic cholangiocarcinoma	2022/9/30	Starting dose: 20 mg po qd First dose reduction: 16 mg Second dose reduction: 12 mg	ORR: 42% N = 103 Median DoR: 9.7 months DoR ≥ 6 months, n (%): 31 (72%) DoR ≥ 12 months, n (%): 6 (14%)	ALL (39%) Pyrexia (3.9%) Gastrointestinal hemorrhage (3.9%) Ascites (2.9%) Musculoskeletal pain (2.9%) Bile duct obstruction (2.9%)	Nail toxicity (47%) Musculoskeletal pain (43%) Constipation (39%) Diarrhea (39%) Fatigue (37%)	Increased phosphate (97%) Increased creatinine (58%) Decreased hemoglobin (52%) Increased glucose (52%) Increased calcium (51%)
Pemigatinib	FGFR2 gene fusions or rearrangements	Unresectable advanced or metastatic cholangiocarcinoma	2020/4/17	Starting dose: 13.5 mg po d1-d14 q3w First dose reduction: 9 mg Second dose reduction: 4.5 mg	ORR: 36% N = 107 CR: 2.8% PR: 33% Median DoR: 9.1 months DoR ≥ 6 months, n (%): 24 (63%) DoR ≥ 12 months, n (%): 7 (18%)	ALL (45%) Abdominal pain (4.8%) Fatigue (4.8%) Ascites (2.9%) Musculoskeletal pain (2.9%) Bile duct obstruction (2.9%)	Alopecia (49%) Diarrhea (47%) Nail toxicity (45%) Fatigue (42%) Dysgeusia (40%)	Increased phosphate (94%) Decreased phosphate (68%) Increased aspartate aminotransferase (43%) Increased calcium (43%) Decreased sodium (39%)
Erdafitinib	FGFR3 or FGFR2 genetic alterations	Advanced or metastatic urothelial carcinomae (at least one line of prior platinum_x0002_containing chemotherapy)	2019/4/12	1. Starting dose: 8 mg po qd First dose reduction: 6 mg Second dose reduction: 5 mg Third dose reduction: 4 mg 2. Starting dose: 9 mg po qd First dose reduction: 8 mg Second dose reduction: 6 mg Third dose reduction: 5 mg Forth dose reduction: 4 mg	ORR: 36% N = 87 CR: 2.3% PR: 29.9% Median DoR: 5.4 months	ALL (67%) Onycholysis (10%) Fatigue (10%) Stomatitis (9.0%) Palmar-plantar erythrodysesthesia syndrome (6%) Dry eye (5.0%)	Stomatitis (56%) Fatigue (54%) Diarrhea (47%) Dry mouth (45%) Decreased appetite (38%)	Increased phosphate (76%) Increased creatinine (52%) Increased aspartate aminotransferase (41%) Decreased Sodium (40%) Decreased hemoglobin (35%)
Infigratinib	FGFR2 gene fusions or rearrangements	Unresectable advanced or metastatic cholangiocarcinoma	2021/5/28 (Delisting announced on October, 2022)	Starting dose: 125 mg po d1-d21 q4w First dose reduction: 100 mg Second dose reduction: 75 mg Third dose reduction: 50 mg	ORR: 23% N = 108 CR: 1% PR: 22% Median DoR: 5.0 months DoR ≥ 6 months, n (%): 8 (32%) DoR ≥ 12 months, n (%): 1 (4%)	Stomatitis (15%) Palmar-plantar erythrodysesthesia syndrome (7%) Abdominal pain (5.0%) Fatigue (4.0%) Diarrhea (3.0%)	Nail toxicity (57%) Stomatitis (56%) Dry eye (44%) Fatigue (44%) Alopecia (38%)	Increased creatinine (93%) Increased phosphate (90%) Decreased phosphate (64%) Increased alkaline phosphatase (54%) Decreased hemoglobin (53%)

### Selective inhibitors

#### AZD4547

AZD4547 is a small-molecule tyrosine inhibitor that selectively targets FGFR 1, 2, and 3 [131]. In the phase II S1400D trial, AZD4547 demonstrated limited efficacy in squamous cell NSCLC patients with *FGFR* alterations, despite its satisfactory safety profile [132]. Similarly, the NCI-MATCH trial revealed restricted effectiveness of AZD4547 in refractory cancers exhibiting *FGFR*1-3 aberrations, with responses observed solely in tumors harboring *FGFR*1-3 point mutations or fusions [133]. The SHINE study findings indicate that AZD4547 does not yield a statistically significant enhancement in PFS when compared to paclitaxel in patients with advanced GC who exhibit *FGFR*2 amplification/polysomy [134]. Meanwhile, These results underscore the presence of intratumor heterogeneity and a lack of concordance between *FGFR*2 amplification and mRNA expression. The RADICAL study discovered that the combination of AZD4547 with anastrozole or letrozole exhibited limited efficacy but notable toxicity in patients with estrogen receptor-positive metastatic breast cancer who had developed resistance to aromatase inhibitors [135]. A subsequent study revealed that AZD4547 exhibited no discernible efficacy among patients diagnosed with malignant pleural mesothelioma who experienced disease progression after initial treatment involving platinum-based chemotherapy [136].

#### HMPL-453

HMPL-453 is a potent inhibitor of FGFR 1, 2, and 3 and exhibits potent antitumor activity in tumor models with FGFR alterations [137]. In previously treated patients with advanced ICC and FGFR fusion, the HMPL-453 (300 mg, QD, 2w on/1w off) dosing arm demonstrated acceptable levels of toxicity and excellent efficacy: an ORR of 50% and a DCR of 90% [138]. Multiple additional studies have been devised to assess the effectiveness, safety, and pharmacokinetics of HMPL-453 in advanced malignant mesothelioma and other advanced solid tumors, as well as to investigate the potential synergistic effects of combining HMPL-453 with chemotherapy or anti-PD-1 antibodies (NCT05173142, NCT04290325, NCT03160833).

#### RLY-4008

RLY-4008 is a selective FGFR2 inhibitor and remains inhibitory to tumors that develop resistance mutations [139, 140]. In a clinical investigation examining the effects of RLY-4008 on patients diagnosed with bile duct cancer characterized by *FGFR*2 fusion/rearrangement, a combined phase I/II trial demonstrated favorable efficacy, as evidenced by an ORR of 88% at the phase II dosage level. The administered treatment exhibited a high level of tolerability, primarily manifesting as low-grade side effects, and notably, no instances of severe (grade 4/5) treatment-related adverse events were recorded. Furthermore, the sustained nature of most responses suggests the promising prospect of a prolonged and enduring therapeutic response [141].

#### Rogaratinib

Rogaratinib, a potent inhibitor of FGFR1-4, exhibited significant inhibition of various tumors characterized by *FGFR* aberrations [142]. In a clinical trial investigating the efficacy of rogaratinib in patients with a variety of solid tumors, 15 of 100 patients had an objective response to treatment (ORR: 15%) [143]. In a separate phase II/III clinical trial conducted on individuals diagnosed with *FGFR* mRNA-positive advanced/mUC, rogaratinib demonstrated comparable levels of effectiveness and safety when compared to chemotherapy, exhibiting similar rates of ORR (20.7% vs 19.3%). Nevertheless, an examination of patients exhibiting *FGFR*3 DNA alterations revealed a substantial disparity in OR (52.4% vs. 26.7%) [144]. Another study demonstrated that despite the elevated expression of *FGFR* mRNA in approximately 50% of tumors in patients with SqCC, the administration of rogaratinib did not result in a significant improvement in PFS among these individuals [145].

#### FGF401

FGF401 is a reversible, covalent, small-molecule inhibitor of FGFR4 kinase activity that has shown significant antitumor efficacy in preclinical studies [146]. The results of the NCT02325739 trial showed that 74 patients treated with single-agent FGF401 in phase I and 86 patients treated with FGF401 in phase II experienced a total of 8 patients with objective responses (1 CR, 7 PR; 4 each in phases I and II), demonstrating some clinical efficacy and safety [147].

#### E7090

E7090 is an FGFR1-3 inhibitor that has shown promise in the treatment of mouse tumor models with FGFR abnormalities [148]. Multiple phase I clinical trials investigating the effects of E7090 in patients with advanced solid tumors revealed a tolerable safety profile, absence of dose-limiting toxicities at doses equal to or exceeding 140 mg, and promising indications of therapeutic efficacy [149, 150]. E7090 has been shown in NCT04238715 Phase II to have strong anti-tumor efficacy (ORR of 30%; DCR of 79%) and to be safe and controlled in the treatment of CCA patients with FGFR2 gene fusion [151].

#### ICP-192

ICP-192, a newly developed covalent inhibitor targeting pan-FGFR, has shown promising results in Phase I clinical trials [152]. A phase IIa dose extension studied the efficacy of ICP-192 in previously treated patients with FGFR2-altered CCA, resulting in an ORR of 52.9% (9/17), an mPFS of 6.93 months, and a discontinuation rate of 0% due to TRAEs, suggesting that ICP-192 had a favorable response rate and was well tolerated [153]. Simultaneously, several Phase II clinical trials are being conducted for the subject (NCT04565275, NCT04492293, NCT05678270, NCT05372120).

### Multi-targeting TKIs

#### Lenvatinib

Lenvatinib, a selective, multi-target tyrosine kinase inhibitor, exerts its action on the vascular endothelial growth factor receptor (VEGFR), FGFR, and platelet-derived growth factor receptor (PDGFR) family [154, 155]. In vitro experiments have provided evidence that lenvatinib effectively suppresses the proliferation signaling pathway of overexpressed VEGFR and FGFR in cancer cells [156]. Interestingly, lenvatinib offers a promising therapeutic option for *FGFR* 2-driven CCA, especially in cases involving insurmountable adverse effects to selective Tkis or acquired kinase mutations [157]. A Phase III clinical trial showed that lenvatinib was not inferior to sorafenib in patients with previously untreated advanced HCC (median survival time: 13.6 vs. 12.3 months), in contrast to sorafenib, lenvatinib exhibited substantial enhancements across all secondary endpoints, including a greater ORR, extended PFS, and increased time to progression (TTP) [158]. A separate investigation demonstrated that lenvatinib exhibited a noteworthy enhancement in both PFS and response rates among individuals afflicted with iodine 131-refractory thyroid cancer [159]. In the cohort of patients diagnosed with advanced renal cell carcinoma (RCC) and who had not undergone previous systemic therapy, the combination of lenvatinib and pembrolizumab demonstrated a significantly extended PFS compared to sunitinib (median, 23.9 months versus 9.2 months) [160]. Similarly, in patients with advanced endometrial cancer after failure of prior platinum-based chemotherapy, lenvatinib combined with pembrolizumab significantly prolonged PFS (6.6 months vs 3.8 months) and OS compared with chemotherapy (17.4 months vs 12.0 months) [161]. According to the research findings, numerous extensive phase III clinical trials are currently investigating the effectiveness of the lenvatinib and pembrolizumab combination [162–165].

#### Surufatinib

Surufatinib, a multi-targeted tyrosine kinase inhibitor, primarily acts on VEGFR1-3 and FGFR1 [166]. In the cohort of individuals diagnosed with advanced neuroendocrine tumors, the duration of PFS was notably extended in those who received surufatinib treatment (9.2 months compared to 3.8 months). Furthermore, among patients with progressive well-differentiated extra pancreatic NETs, surufatinib demonstrated a favorable benefit-risk ratio [167].

#### Nintedanib

Nintedanib, a derivative of indolinone, has demonstrated the ability to effectively inhibit the kinase activity of VEGFR, PDGFR, and FGFR in the assay [168]. This compound has been identified as a specific therapeutic agent for the treatment of pulmonary fibrosis and has shown promising results in preclinical studies by effectively suppressing the growth of diverse tumor types [169–171]. According to the findings of a phase III clinical trial, the combination of nintedanib and docetaxel has demonstrated efficacy as a second-line treatment for patients with advanced NSCLC, particularly those with adenocarcinoma, who have previously undergone platinum-based chemotherapy. Following a median follow-up period of 31.7 months, the median OS was determined to be 12.6 months for the nintedanib plus docetaxel group, compared to 10.3 months for the placebo plus docetaxel group [172]. Nevertheless, despite its high tolerability, nintedanib did not yield a substantial advantage in individuals with refractory colorectal cancer following the ineffectiveness of conventional treatment [173]. Similarly, nintedanib did not confer a significant benefit as a maintenance therapy in patients with malignant pleural mesothelioma who had previously received pemetrexed plus cisplatin [174].

#### Anlotinib

Anlotinib is an innovative orally administered tyrosine kinase inhibitor that selectively targets VEGFR, FGFR, PDGFR, and c-kit [175]. In a phase III randomized clinical trial in a Chinese cohort of patients with advanced NSCLC, the administration of anlotinib demonstrated a notable extension in both OS (9.6 months vs. 6.3 months) and PFS (5.4 months vs. 1.4 months) [176]. In another phase II clinical trial, a significant proportion of patients (56.9%) diagnosed with unresectable locally advanced or metastatic medullary thyroid cancer and subjected to anlotinib treatment exhibited a partial response. Furthermore, the PFS rate after 48 weeks of treatment was reported to be 85.5% [177]. A separate study was conducted to assess the effectiveness and safety of anlotinib as a primary treatment for metastatic renal cell carcinoma. The findings indicated that anlotinib exhibited comparable efficacy to sunitinib, as evidenced by similar median PFS durations (17.5 months vs. 16.6 months), median OS durations (30.9 months vs. 30.5 months), and ORR (30.3% vs. 27.9%). Furthermore, anlotinib demonstrated superior safety profiles when compared to sunitinib [178]. In the third-line or subsequent treatment of patients with small cell carcinoma, anlotinib showed better PFS and OS compared with placebo, while maintaining a favorable safety profile [179]. In patients with advanced or metastatic HCC who were previously treated with a tyrosine kinase inhibitor, the 12-week PFS rate and median TTP were 72.5% and 4.6 months, respectively, showing favorable efficacy [180].

#### Lucitanib

Lucitanib, a small molecule inhibitor targeting VEGFR1-3, PDGFRα/β, and FGFR1-3 tyrosine kinases, exhibited notable suppression of tumor growth across diverse xenograft models owing to its robust angiogenesis inhibition [181]. When used in conjunction with fulvestrant, Lucitanib effectively impeded the proliferation of ER+/*FGFR*1-amplified cells and patient-derived xenografts (PDX) [182]. The findings of a phase II study revealed that among patients diagnosed with HR+/HER2(−) metastatic breast cancer and who had undergone no more than one prior chemotherapy treatment, the group with *FGFR*1 amplification (consisting of 32 individuals) exhibited an ORR of 19% when treated with Lucitanib [183].

#### Derazantinib

Derazantinib, a novel multi-kinase inhibitor, shows potent activity against *FGFR*-addicted cell lines and tumors [184], and in models of GC driven by FGFR, derazantinib exhibited greater efficacy compared to paclitaxel [185]. In a phase I/II clinical trial, derazantinib exhibited encouraging anti-tumor efficacy and tolerable safety profiles among patients diagnosed with advanced, unresectable ICC harboring *FGFR*2 fusion, resulting in a notable response rate of 20.7% and a disease control rate of 82.8% [186]. Preliminary data analysis from a comparable study additionally indicates that the administration of derazantinib yields favorable clinical outcomes among patients with advanced ICC harboring *FGFR*2 aberrations [187]. Regrettably, despite demonstrating certain efficacy in select patients with mUC and *FGFR*1-3 gene alterations, the observed ORR and PFS did not meet the required benchmarks to substantiate the continuation of derazantinib as a standalone therapeutic approach for this indication [188].

#### Tinengotinib

Tinengotinib is a multi-kinase inhibitor that strongly inhibits Aurora A/B, FGFR1/2/3 and VEGFRs, as demonstrated in kinase assays. In both in vitro and in vivo studies, exposure to tinengotinib specifically suppressed the proliferation of all subtypes of triple-negative breast cancer while preserving the integrity of luminal breast cancer cells [189]. Qualified patients with advanced/metastatic CCA who had exhausted the standard treatment options received tinengotinib 10 mg once daily (QD). In a cohort with acquired resistance to FGFR inhibitors, 2 out of 6 patients (33%) achieved PR with tumor reduction of 34% and 54%, respectively. The overall disease control rate (DCR, including CR or PR + stable disease (SD)) for *FGFR*2 fusion/rearrangement patients was 90% (9/10) [190].

### Monoclonal antibody

#### Vofatamab

MFGR1877A is a human IgG1 monoclonal antibody (mAb) that can bind to FGFR3 [191]. In the mUC population with prior 1st-line chemotherapy failure or relapse, the combination strategy of vofatamab combined with docetaxel resulted in a < 40% incidence of disease progression in the patient population; when combined with pembrolizumab, the ORR was 30%, and both treatment strategies were well tolerated [192, 193].

#### Bemarituzumab

Bemarituzumab, a humanized immunoglobulin G1 mAb, specifically targets FGFR2b overexpression in certain tumors. It exhibits a dual mechanism of action, involving the inhibition of FGFR2b signaling and the enhancement of antibody-dependent cell-mediated cytotoxicity (ADCC) [194]. In the phase I trial (FIGHT), patients with HER2-negative, FGFR2b-selected gastric or gastro-esophageal junction adenocarcinoma demonstrated promising clinical efficacy following combination therapy with Bemarituzumab and mFOLFOX6 [195]. After 24 months of follow-up, patients with FGFR2b-overexpressing gastric or gastro-esophageal junction adenocarcinoma treated with bemarituzumab + mFOLFOX6 continued to exhibit clinically meaningful outcomes compared to those treated with placebo + mFOLFOX6 (Median OS: 19.2 months vs. 13.5 months) [196]. Related phase III clinical studies are currently underway [197].

### Drug combination therapies

The utilization of multiple anticancer agents in oncology, known as combination drugs, is a strategy aimed at enhancing therapeutic efficacy. By targeting distinct pathways in cancer cells, these combinations can prevent drug resistance, improve effectiveness, and potentially reduce adverse effects. In the application of FGFR inhibitors, there also seems to be surprising prospects for the use of combination drugs.

### Combination with chemotherapy drugs

Targeted agents are frequently administered in conjunction with chemotherapy in clinical settings to optimize therapeutic efficacy by selectively targeting molecular pathways while simultaneously utilizing the broad cytotoxic effects of chemotherapeutic agents. A prime illustration of this approach is the administration of trastuzumab in combination with docetaxel for HER2-positive breast cancer [198], which leverages complementary mechanisms of action to attain superior therapeutic outcomes.

The incorporation of anlotinib into the standard etoposide/platinum chemotherapy regimen demonstrates promising PFS and OS outcomes in individuals with previously untreated extensive-stage small cell lung cancer [199]. Its combination with capecitabine and oxaliplatin also showed considerable ORR, DCR, PFS and DOR in the first-line treatment of metastatic colorectal cancer [200]. Presently, an ongoing clinical trial (KY20192111-F-1) is being conducted to evaluate the efficacy of anlotinib in conjunction with the SOX regimen for the treatment of stage IV GC [201]. Nevertheless, when combined with diverse chemotherapeutic agents, the outcomes yielded synergistic, additive, and antagonistic effects, respectively. This implies that the combination approach may hold promise for the treatment of these tumors, but additional research is necessary [202, 203].

### Combination with other targeted therapies

The concomitant use of FGFR inhibitors with other targeted therapies has the potential to augment efficacy by addressing tumor heterogeneity and multiple aberrant signaling pathways concurrently [204]. Such combinations may yield synergistic effects and overcome resistance mechanisms commonly encountered with monotherapy, owing to their ability to target diverse pathways. For example, FGFR inhibitors have been used in combination with VEGF/VEGFR inhibitors to combat tumors by a mechanism that inhibits a key process in tumor growth and metastasis: angiogenesis [205]. Other than that, FGFR inhibitors are also used with poly-ADP ribose polymerase (PARP) inhibitors in patients with refractory solid tumors, and preliminary results show that this regimen has an acceptable safety profile [206]. While these combinations have shown some promise, it is important to note that their effectiveness and safety will vary depending on the individual patient's condition and other factors.

### Combination with ICB therapies

Preclinical studies have shown that FGF/FGFR signaling is involved in the regulation of the tumor microenvironment (TME), including immune cells, angiogenesis, and epithelial-mesenchymal transition (EMT) [207]. This makes the application of ICB in combination with FGFR tyrosine kinase inhibitors possible [208–210].

FGFR-TKI targets FGFR4 to increase the proteasomal degradation of PD-L1, inhibits STAT5 phosphorylation to prevent Treg development, and restores the sensitivity of HCC cells to T cell death [211]. Of note, in a trial of erdafitinib, patients with previous ICB therapy had a higher response rate compared with the entire cohort (59% vs. 40%) [212]. The efficacy of combination treatments has also been supported in real-world studies, in breast cancer patients, 60% of the combination therapy group achieved complete remission, accompanied by a significant increase in CD4 + and CD8 + T cell infiltration [213]. Sintilimab combined with anlotinib is effective and safe as second-line treatment for patients with advanced cervical cancer and endometrial cancer who failed previous chemotherapy [214, 215]. The combination of Lenvatinib with anti-PD-1 therapy also leads to the formation of long-term immune memory, while acting synergistically to regulate the normalization of TME and tumor vasculature and to enhance the cytotoxic effects of T cells, improving the efficacy against HCC [216]. Many clinical studies underway based on positive results from preclinical studies. Even though conclusive results are yet to be disclosed in most studies, several clinical studies have provided preliminary validation of the safety of this combination therapy [217–220]. However, data from some clinical studies have shown that FGFR-ICB combined therapy did not achieve significant benefits [221]. FGFR3 has been found in previous studies to be associated with a diminished response to ICBs, however, results from two phase II clinical trials Checkmate 275 and IMVigor 210 showed no statistically significant difference in ICB treatment response rate or OS between patients with or without *FGFR*3 mutations [222]. The researchers posit that this result could potentially be influenced by a confluence of factors, including modifications to the tumor microenvironment, genetic variations among patients, changes in drug resistance, variations in tumor staging, and specific biomarkers [6, 40, 210, 223].

In summary, while preclinical investigations have demonstrated the potential of FGFR in combination with other therapeutic modalities to elicit antitumor effects, this observation has been validated only in a limited number of malignancies in clinical settings, with no direct outcomes observed in other tumor types.

## Other potential treatments

### Antibody drug conjugates

Antibody–drug conjugates (ADCs) represent an innovative class of medications that combine monoclonal antibodies, toxin payloads, and linkers to achieve targeted cancer therapy. ADC drugs leverage the high specificity of monoclonal antibodies to target overexpressed antigens on tumor cells. The linker connects the antibody to the toxin, which is then delivered into the tumor cells under the guidance of the antibody. AMB302/GR1017, an ADCC derived from FGFR3-targeting antibody AimedBio conjugated with topoisomerase-1 inhibitor TopoIx, exhibits potent antitumor efficacy in glioblastoma and bladder cancer models with *FGFR*3 amplification or *FGFR*3-*TACC*3 fusion, both in vitro and in vivo [224]. It notably demonstrates significant antitumor activity against glioblastoma patient-derived cells (PDCs) in an *FGFR*3-*TACC*3-dependent manner, surpassing the performance of similar antibodies and Deruxtecan as payload for ADC drugs. Moreover, the compound extends the survival time of the *FGFR*3-*TACC*3 fusion glioblastoma orthotopic PDX model by 200% and induces complete tumor regression in the *FGFR*3-*TACC*3 fusion RT112 BC model. Additionally, AMB302/GR1017 shows excellent tolerability in rodent models, even at therapeutic doses up to 200 mg/kg, reinforcing its potential as a well-tolerated and effective treatment option for these malignancies.

### FGF traps

A heterogeneous group of molecules known as FGF traps possess the ability to function as FGFR decoys. These molecules bind to FGFs in the extracellular environment, effectively preventing the growth factors from interacting with target cells. An example is FP-1039, an FGF ligand capture agent incorporating the extracellular domain of the FGFR1-IIIc splice isoform. A recent phase I study involving patients with metastatic or locally advanced solid tumors, treated with FP-1039, revealed the most favorable response to be SD (41.7%) among the 39 patients studied [225]. Notably, no discernible relationship between abnormalities in the FGF pathway and the observed antitumor effects was identified.

### Radionuclide-conjugated drugs

Radionuclide-conjugated drugs are a category of therapeutic agents that merge radionuclides with distinct drug molecules. The fundamental concept underlying nuclide-conjugated drugs is to guide radionuclides to specific treatment sites, where radiation is emitted through radioactive decay, inducing a lethal impact on targeted cells. [225Ac]-FPI-1966 represents a therapeutic α-emitting drug with FGFR-targeting properties. This compound merges the FGFR3 monoclonal antibody vofatamab with the α-emitting radionuclide Actinium-225. Currently, it is the subject of investigation within a Phase I/II study focused on evaluating FGFR3 expression (NCT05363605).

### FGFR-TKI resistance in tumors

FGFR-TKI resistance in tumors refers to the phenomenon where tumors become unresponsive to treatment with tyrosine kinase inhibitors targeting the FGFR pathway. This resistance can arise through various mechanisms, such as genetic mutations in the FGFR gene, activation of alternative signaling pathways, or adaptive cellular responses [226]. Such resistance complicates treatment and necessitates the development of novel therapeutic strategies to overcome or circumvent it, ensuring continued efficacy of anti-cancer therapies in patients with FGFR-driven tumors.

### FGFR gatekeeper mutations

Resistance to FGFR inhibitors mainly originates from mutations in the targeted kinase, especially mutations in the “gatekeeper” residues [6, 227–231]. The access control residues are in the hinge region connected to the C-terminal end of the kinase structural domain and have an important function in modulating the availability of hydrophobic pockets [232, 233]. Any mutation in these residues may stabilize the active kinase conformation by detaching the molecular brake or enhancing the hydrophobic spine or mimicking the action of A-ring tyrosine phosphorylation, thereby enhancing resistance to inhibitors [234]. The occurrence of a gatekeeper mutation has the potential to disrupt the crucial hydrogen bonds necessary for establishing strong affinity interactions or induce three-dimensional clashes that impede the binding of inhibitors [235–238]. All FGFR kinases have a threonine gatekeeper residue at the pathway control position, and mutations in this residue can lead to resistance to a generation of FGFR-TKIs [230]. Considering this alteration, LY2874455 undergoes a conformational change resembling that of a chair, resulting in the folding of the hydrophobic residue Leu619 in *FGFR*4V550L/M [239]. This conformational property results in an increased spatial distance between the mutant amino acid residue and the compound, effectively bypassing resistance by avoiding collision with the mutant gatekeeper residue. The covalent inhibitor TAS-120 possesses a flexible connector within its core structure. This feature allows the dimethoxybenzene ring of TAS-120 to exhibit significant rotational flexibility [240]. Consequently, the inhibitor can adapt to the hydrophobic pocket of FGFR, thereby overcoming the gatekeeper mutation. In addition, several novel 3-aminopyrazole compounds take the approach of covalently modifying p -cyclic cysteine residues in response to gatekeeper mutations in FGFR2 and FGFR3 [230, 241].

### Alternative activation associated with the FGFR pathway

Cancer cells show remarkable flexibility in controlling their proliferation in terms of RTKs, often exhibiting crosstalk between signals with alternative activation [242, 243]. This convergence can result in the activation of additional downstream signals through the process of cross-phosphorylation of RTKs. Preclinical research suggests that in cases of acquired resistance to infigratinib, the AKT pathway experiences positive regulation [244, 245]. In addition, the interaction between FGFR and IGFR prevents complete inhibition of MAPK signaling by inhibiting FGFR, which is associated with intrinsic and acquired resistance to FGFR inhibitors [246]. In *FGFR* amplification tumors, co-activated RTKs ligands, including EGFR family proteins and MET, serve as alternative RTKs. These alternative RTKs are associated with *FGFR*2 amplification and confer resistance to FGFR inhibitors in FGFR3 translocation cells [243, 247]. The activation of AKT and ERK signals by MET was facilitated by GAB1, and the intercommunication of signals mediated by GAB1 resulted in the development of resistance to FGFR inhibitors in the cell line [246]. The concurrent administration of MET inhibitors and FGFR inhibitors has demonstrated a potent ability to effectively reverse this phenomenon [243]. The activation of EGFR serves as the mechanism by which PD173074 treatment is evaded. The co-administration of Gefitinib and PD173074 demonstrates the potential to surmount drug resistance in urothelial cancer cases characterized by *FGFR*3 molecular alterations [247]. Src, HER2 and EphB3 pathways serve as alternative resistance mechanisms in uroepithelial cancer cells and gastric cancer, respectively, and can be overcome by combining FGFR-TKIs with corresponding inhibitors [226, 248–250]. The PROTAC drug LC-MB12 developed by FGFR has the potential to interfere with FGFR by inducing asymmetric dephosphorylation dimerization, thereby disrupting the contact points of downstream signaling molecules, including the FRS2/SHP2/Gab1/Grab2 complex [251, 252]. To mitigate the scaffolding effect of FGFR2 and impede the advancement of the compensation feedback pathway.

### Lysosomal isolation

Lysosomes comprise of lipoprotein membranes, serving as digestive vesicles. They have acidic hydrolases that eliminate excess macromolecules like proteins, nucleic acids, lipids and polysaccharides from cells [253, 254]. The lysosomal environment is acidic, hence weakly basic FGFR kinase inhibitors become protonated and cannot recross the lysosomal membrane, thus being trapped in lysosomal vesicles [254]. This process necessitates the cooperation of the ABC transporter and is controlled by affirmative feedback from TFEB (TFEB is a transcription factor involved in the regulation of lysosomal and autophagic systems in cells)-induced lysosome production [255–258]. The intracellular gathering and distribution of nintedanib and PD173074 are demonstrated through three-dimensional fluorescence spectroscopy and other techniques that affirm their resistance mediated by lysosomes [259, 260]. Additionally, autophagy is closely linked to lysosome mediated TKI resistance [261]. Activation of the mTOR signaling pathway triggers autophagy to support survival against drug treatment in FGFR-TKI-resistant GC cell lines, and TAK1 aggravates this process. A synergistic therapeutic approach involving NG25 (a TAK1 inhibitor) and AZD4547 can reverse this phenomenon. Modifying the structure of the TKI, interfering with the lysosome's typical functioning and structure, and obstructing the ABC transporter and kinase are deemed to be efficacious techniques in conquering lysosome-mediated resistance [262–265].

### The side effects of FGFR-inhibitors and the mechanism

**Fig. 5:**
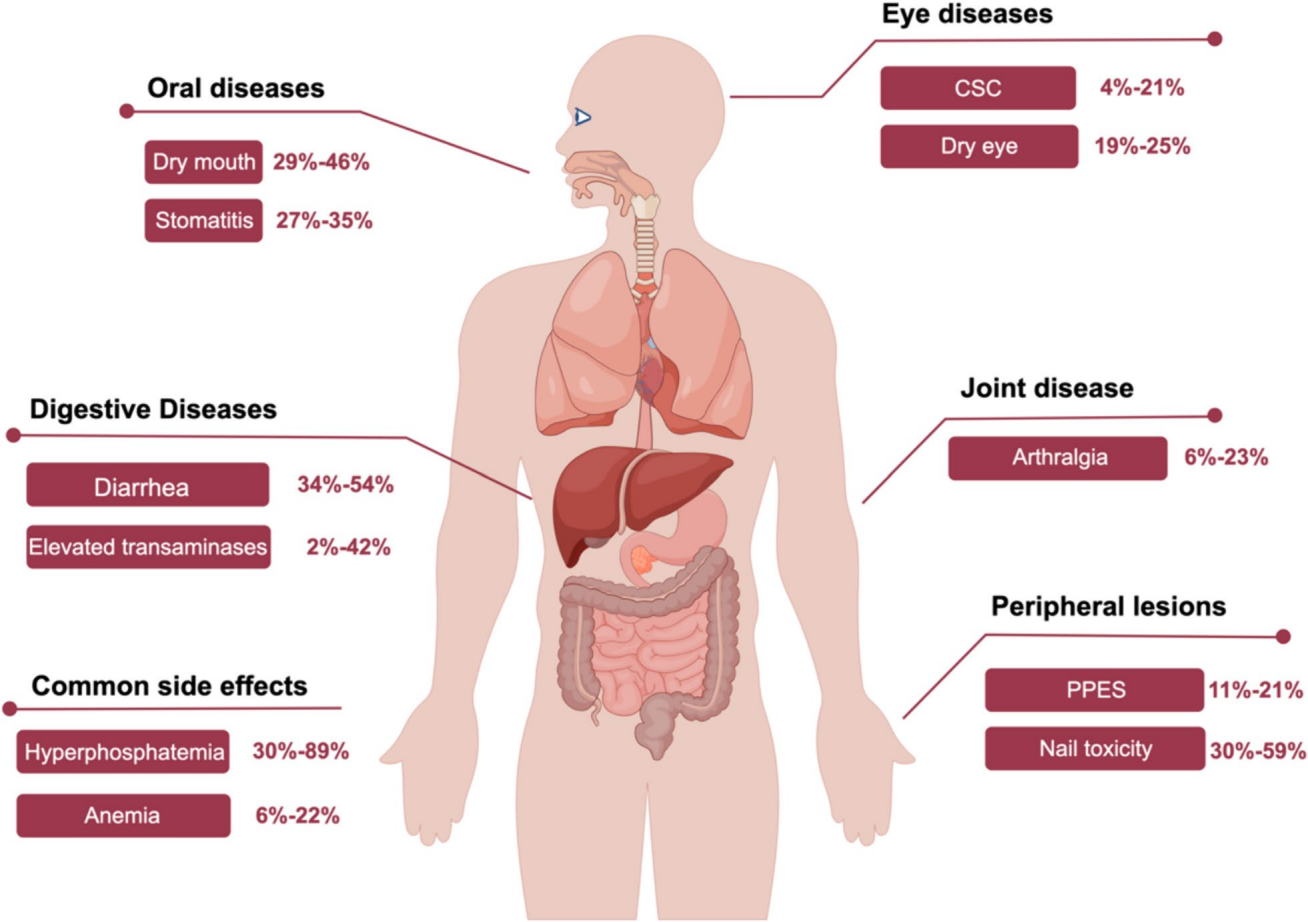
Common side effects of FGFR inhibitor. The side effects of FGFR inhibitors can be seen in many systems throughout the body, among which hyperphosphatemia is the most common. (CSC: Central plasmacytoid choroidal retinopathy; PPES: palmar-plantar erythrodysesthesia syndrome)

With any therapeutic agent, there must be a trade-off between efficacy and toxicity to optimize clinical benefit. Common targeted toxicities associated with FGFR inhibitors include hyperphosphatemia and nail, skin, and ocular toxicity [128, 212]. However, there is still an unmet need for practical insights into AE management in the real world (Fig. 5).

Hyperphosphatemia is one of the most reported AEs in clinical trials of FGFR inhibitors, with hyperphosphatemia reported in no less than 60% of patients in the BLC2001, FIGHT-202, and FOENIX-CCA2 trials [109, 117, 128]. FGFR1 is normally involved in the regulation of phosphorus metabolism in vivo, and when FGFR1 is inhibited, this regulation is disturbed, resulting in elevated serum phosphorus levels, which is thought to be a targeted FGFR inhibitor-like effect [266]. In most cases, hyperphosphatemia can be alleviated with appropriate management. In trials such as FIGHT-202 and FOENIX-CCA2, no more than 2% of patients were discontinued or had a change in dose after being placed on a low-phosphorus diet and given phosphate binders and diuretics [109, 117, 128].

Similarly, a 30–59% incidence of nail toxicity and an 11–21% incidence of palmar-plantar erythrodysesthesia syndrome (PPES) were reported in the BLC2001, FIGHT-202, and FOENIX-CCA2 trials [109, 117, 128]. The pathologic mechanism behind this adverse event has not been elucidated and may be the induction of hair follicle homeostasis dysregulation and epidermal proliferation and/or differentiation by inhibition of FGFR in keratin-forming cells, with concomitant down-regulation of tight junction gene expression [267]. Oral antibiotics against infection, partial or total nail removal, topical urea and cortisol creams or interruption/discontinuation of treatment are considered mandatory in some cases [268].

In the BLC2001 and FIGHT-202 studies, dry eye occurred in 19–25% of patients and central plasma retinopathy in 4–21% [109, 117]. In contrast, ocular side effects occurred less frequently in the FOENIX-CCA2 trial, with dry eye occurring in approximately 17% of patients [92]. Retinopathy is commonly associated with MAPK pathway inhibitors, and it so happens that FGFR inhibitors can interfere with downstream MAPK-mediated signaling, which is thought to be a potential mechanism for FGFR inhibitor-associated retinopathy [269, 270]. Therefore, it is recommended that a comprehensive ophthalmologic examination be performed prior to the initiation of FGFR inhibitor therapy and during the first few months of treatment, and that dose adjustments be made to control the associated targeting toxicity to ensure adherence.

Treatment-related side effects of FGFR inhibitors are not uncommon. Therefore, active monitoring during treatment is possible to minimize dose reductions and discontinuations and may be beneficial to patients' quality of life and outcomes.

## Conclusions and prospects

FGFR abnormalities are often seen in several forms of cancer, such as bladder, cholangiocarcinoma, gastric cancer, and lung cancer. Presently, both the NCCN and ESMO recommendations endorse the use of FISH, RT-PCR, and NGS techniques to identify this anomaly in cholangiocarcinoma and bladder cancer. The FDA has granted approval for Infigratinib, Pemigatinib, and Futibatinib in the treatment of cholangiocarcinoma with FGFR2 fusion, as well as Erdafitinib in the treatment of bladder cancer with FGFR3 mutation. These medications have shown good clinical effectiveness. Additionally, clinical studies are being conducted for medications such as AZD4547, HMPL-453, and RLY-4008.

Certainly, there remain some inquiries that merit contemplation and investigation. First, is it worth expanding FGFR-targeted therapy clinical indications? *FGFR*1 amplification in 12.5% of breast tumors makes ER + breast cancer patients resistant to CDK4/6 inhibitors and endocrine treatments, increasing their likelihood of disease recurrence [42, 271, 272]. Therefore, appropriate clinical trials could be attempted to improve the clinical prognosis of such patients. Furthermore, there is potential for improvement in the techniques used to identify the specific subset of the population that has the most favorable response to FGFR-TKI. In the FORT-1 trial, patients with FGFR1-3 mRNA overexpressing uroepithelial carcinoma treated with rogaratinib had similar benefits as those treated with chemotherapy [273]. However, in the subgroup that also had altered FGFR3 DNA levels, the ORR increased to 52% compared to 27% in the chemotherapy group [273]. Does this disparity imply that FGFR3 DNA changes serve as a more precise biomarker compared to mRNA overexpression? Alternatively, does it imply that doctors should consider both FGFR3 DNA changes and mRNA overexpression when determining whether to provide FGFR-TKI treatment to a patient? With the help of AI tools, it may be possible to identify the presence or absence of FGFR changes on HE slides, and the results showed that the area under the receiver operating curve value was 0.76 [274]. Each of these methods needs to be validated in a larger cohort. At present, it is uncertain whether patients with urothelial carcinoma with *FGFR* alterations can benefit from ICI, and more relevant exploration is needed. In cohort 1 of the phase III THOR trial, OS and PFS were also significantly longer in urothelial carcinoma patients treated with previous ICI with erdafitinib than in those treated with chemotherapy [275]. However, in cohort 2 of the THOR trial, OS was very similar in patients treated with erdafitinib or pembrolizumab [120]. These differences may reflect increased numbers of immunosuppressive macrophages and regulatory T cells, as well as reduced infiltration of inflammatory CAF and T cells in the tumor microenvironment of cancer patients with *FGFR*3 alterations [276, 277]. Currently, studies are underway to assess the safety and initial effectiveness of combining ICI with FGFR inhibitors [278]. Nevertheless, the existing combination regimens primarily rely on anti-PD-1 therapy, and the combination with other immune checkpoint inhibitors like CTLA4 lacks substantiating evidence. Finally, the emergence of drug resistance during the treatment of cancer patients is an unavoidable topic. There are many mechanisms for the development of drug resistance, among which the most common is the *FGFR* gatekeeper mutation [226]. In addition, the activation of some related pathways, such as *FGFR*2 N550H mutation, which can up-regulate the PI3K/AKT/mTOR signaling pathway, is also associated with drug resistance [279]. Several new mutation-targeting agents have emerged, but they still have some limitations [125, 190]. However, the correlation between the development of resistance to FGFR inhibitors and the microenvironment remains unclear. For patients with FGFR inhibitor resistance, whether the therapeutic effect of combined immunotherapy can be restored or improved is also a topic worth exploring.

## Data Availability

Not applicable.

## Abbreviations

BLCA: Bladder Cancer
BMP: Bone Morphogenetic Protein
CCA: Cholangiocarcinoma
CHOL: Cholangiocarcinoma
CKD: Chronic Kidney Disease
CR: Complete Response
CSC: Central Plasmacytoid Choroidal Retinopathy
ctDNA: Circulating Tumor DNA
DMG: Diffuse Midline Gliomas
ESCC: Esophageal Squamous Cell Carcinoma
FDA: Food and Drug Administration
FGF: Fibroblast Growth Factor
FGFR: Fibroblast Growth Factor Receptor
FGFRL1: Fibroblast Growth Factor Receptor-Like 1
FISH: Fluorescence In Situ Hybridization
GBM: Glioblastoma
GC: Gastric Cancer
HCC: Hepatocellular Carcinoma
HNSCC: Head and Neck Squamous Cell Carcinoma
HS: Heparan Sulfate
HSPG: Heparan Sulfate Proteoglycans
ICC: Intrahepatic Cholangiocarcinoma
LGG: Low-grade Glioma
MKP3: Mitogen-Activated Protein Kinase Phosphatase 3
MM: Multiple Myeloma
MNGT: Mixed Neuronal-Glial Tumors
mUC: Metastatic Urothelial Carcinoma
NGS: Next-Generation Sequencing
NMIBC: Non-Muscle Invasive Bladder Cancer
NSCLC: Non-Small Cell Lung Cancer
ORR: Objective Response Rate
OS: Overall Survival
PAAD: Pancreatic Cancer
PFS: Progression-Free Survival
PI3K/AKT: Phosphoinositide 3-Kinase/Protein Kinase B
PLCγ: Phospholipase C Gamma
PPES: Palmar-Plantar Erythrodysesthesia Syndrome
RTKs: Receptor Tyrosine Kinases
RT-PCR: Reverse Transcription-Polymerase Chain Reaction
SCLC: Small Cell Lung Carcinoma
SEF: Similar Expression to FGF
Spry: Sprouty
SqCC: Squamous Cell Lung Cancer
STATs: Signal Transducers and Activators of Transcription
TKI: Tyrosine Kinase Inhibitor
UCEC: Uterine Endometrial Carcinoma
Wnt: Wingless/Integrated
XFLRT3: Xylosyl transferase 3
